# Enhanced Water Stability and Photoresponsivity in Metal-Organic Framework (MOF): A Potential Tool to Combat Drug-resistant Bacteria

**DOI:** 10.1038/s41598-019-55542-8

**Published:** 2019-12-18

**Authors:** Saleh A. Ahmed, Damayanti Bagchi, Hanadi A. Katouah, Md. Nur Hasan, Hatem M. Altass, Samir Kumar Pal

**Affiliations:** 1 Chemistry Department, Faculty of Applied Sciences, Umm Al-Qura University, 21955 Makkah, Saudi Arabia; 2 Chemistry Department, Faculty of Science, Assiut University, 71516 Assiut, Egypt; 3 Department of Chemical, Biological and Macromolecular Sciences, S. N. Bose National Centre for Basic Sciences, Block JD, Sector III, SaltLake, Kolkata 700 106 India

**Keywords:** Metal-organic frameworks, Organic-inorganic nanostructures

## Abstract

In this work, we have successfully synthesized a bimetallic (Zinc and Cobalt) Zeolitic Imidazolate Framework (Zn_50_Co_50_-ZIF), a class in a wider microporous Metal-Organic Framework (MOF) family. The synthesized nanostructures maintain both water stability like ZIF-8 (solely Zn containing) and charge transfer electronic band in the visible optical spectrum as ZIF-67 (solely Co containing). Crystal structure from XRD, high resolution transmission electron microscopy (HRTEM) followed by elemental mapping (EDAX) confirm structural stability and omnipresence of the metal atoms (Zn and Co) across the nanomaterial with equal proportion. Existence of charge transfer state consistent with ZIF67 and intact ultrafast excited state dynamics of the imidazolate moiety in both ZIF-8 and ZIF-67, is evidenced from steady state and time resolved optical spectroscopy. The thermal and aqueous stabilities of Zn_50_Co_50_-ZIF are found to be better than ZIF-67 but comparable to ZIF-8 as evidenced by solubility, scanning electron microscopy (SEM) and XRD studies of the material in water. We have evaluated the photoinduced ROS generation by the mixed ZIF employing dichloro-dihydro-fluorescein diacetate (DCFH-DA) assay. We have also explored the potentiality of the synthesized material for the alternate remediation of methicillin resistant *Staphylococcus aureus* (MRSA) infection through the photoinduced reactive oxygen species (ROS) generation and methylene blue (MB) degradation kinetics.

## Introduction

Treatment of antibiotic resistant infections is a global hazard to human civilization^1^ poses an open challenge to the contemporary therapeutic research to combat multi-drug resistant bacteria^2,3^. In this direction, nanomaterials have emerged as a substitute tool to fight multi-drug resistant bacteria^4^. As the nanomaterial-bacteria interaction relies on several physical phenomena namely electrostatic attraction, van der Waals forces and hydrophobic nature, a profound scientific understanding of the interaction, which may not be unique for all cases provides ultimate insight for designing novel antimicrobial agents^5^. The key factor for the development of new antibiotics using nanomaterials is unique according to its physiochemical properties, which essentially offers a versatile platform to develop innovative therapeutic strategies^6^. In general, the therapeutic strategies using nanomaterials can be classified into two categories namely direct and indirect interaction of the nanometals with target microorganisms. The direct strategy involves development of a class of organic-inorganic nanohybrid, which either included or directly interacts with the microorganisms till their deactivation. For example, in a recent study vancomycin-functionalized gold nanoparticles (AuNPs) were synthesized to overcome vancomycin-resistant enterococci (VRE)^7,8^. The improved antibacterial activity of the nanohybrids attributed to the enhanced internalization of the antibiotics insidecells connected to the polyvalent effect of concentrated antibiotics present on the surface of nanomaterials^9^. The mechanism of the bactericidal effect was reported to be disruption of negatively charged cell membrane of the bacteria by the cationic polymer and eventually release of Ag^+^ ions from the AgBr nanoparticles^10^. In addition, biopolymers such as DNA, proteins/antibodies are also used in order to develop nanohybrids with specific recognition properties^11^. Antibacterial activity of DNA stabilized Ag nanoparticles was demonstrated by varying the oligonucleotide sequence^12^. It was concluded that the structure of the DNA used to stabilize the nanoparticles plays a key role in cellular internalization and eventually antimicrobial activity^12^.

The indirect antimicrobial effect can be achieved through the use of the nanomaterials as antimicrobial delivery vehicles to successfully deliver them to the infection site^13,14^. In order to qualify for delivery vehicle, a nanomaterial is essential to have following properties, which can only be achieved through careful nano-surface engineering. Firstly, solubility in the bloodstream of the nanomaterial assuring invisibility in opposition to the body’s common defense system as mononuclear phagocytic system can eliminate these nano-vehicles from the bloodstream. Secondly, ability to cross biological barriers through the process of opsonization. As opsonin proteins in blood rapidly adhere to nanomaterials, allowing the macrophages from the MPS (mononuclear phagocytic system) to bind and remove the nanomaterial from circulation^15^. Recently, nitrite-loaded silane-hydrogel based composite nanomaterial was developed to attach the antimicrobial activity of nitric oxide (NO)^16^. Among other nanomaterial-based drug delivery vehicles for pH-Triggered drug delivery systems, enzyme-sensitive delivery systems, bacterial toxin-triggered drug delivery, the strategy for the stimuli-mediated cargo release is both sophisticated and complex, requiring biocompatible materials that can undergo chemical or structural changes in response to stimuli, e.g. light exposure at infection sites^4^. The acidic microenvironments chronic infections, or wounds pH value in the range of 4–7 might be exploited to construct pH-sensitive nanotherapeutics^17^. The exceptional dissolution of ZnO (zinc oxide) NPs nanoparticles at lower pH inducted its useful purpose as nanocarriers for drug delivery applications^18^. Recently, ZnO NPs of great biocompatibility loaded with NIR active photosensitizer are shown to provide unprecedented antimicrobial photodynamic therapeutic efficacy^19^. However, a photo-responsive nano-vehicle for the delivery of conventional drugs, where the delivery vehicle itself shows antibacterial property to drug-resistant bacteria is sparse in the literature and development of such multifunctional nano-material is the motive of the present work.

Metal-organic frameworks (MOFs) constitute a significant class of hybrid inorganic-organic crystalline porous materials and its structures can be manipulated at the atomic scale by an appropriate choice of metal ions and organic ligand^20,21^. MOF shows range of applications in different fields including gas separation, gas storage, catalysis and sensing due to their large surface areas and tunable pore sizes^22–24^. MOFs possess many advantages for the adsorption and release of biomolecules and have a great potential for using as a new age drug-delivery agent. Recently, it has been reported that porous iron(III)-based MOF (MIL) with engineered cores and surfaces able to function as exceptional nanocarriers for powerful controlled delivery of challenging anti-tumoral drugs against cancer^25^. However, poor bioavailability (solubility, stability) under aqueous conditions of most of the MOF systems hindered its use as drug-delivery agent^26^.

Zeolitic imidazolate frameworks (ZIF), a sub-class of MOF, consist of transition metal ions (Zn^2+^ or Co^2+^) and imidazolate linkers forming 3D tetrahedral frameworks frequently like zeolite topologies^27^. Depending upon the metal ions and substitution present at the imidazolate linker, ZIF sometime exhibits exceptional thermal, chemical and water stability. Recently, ZIF-8 co-encapsulating vancomycin and folic acid are shown to have destroyed multi drug-resistant bacterial infection where ZIF-8 acts as active drug delivery agent^28^.

MOFs encompassing its own antibacterial property are mainly reported in the case of Ag containing MOF systems. Recently, it has been reported that Ag containing Zr based MOFs synthesised via two-step process can kill gram-negative *E. coli* infection^29,30^. However, stability and expense of silver are a matter of concern. Cobalt based MOFs are suggested as the only alternatives of Ag-MOF. Co-based MOF using tetrakis[(3,5-dicarboxyphenyl)-oxamethyl] methane acid as ligand has depicted its potency toward inactivation of *E. coli*^31^. Moreover, Co-SIM-1 and ZIF 67 are also reported as potential antibacterial agents. The main issue of Co-based MOF is its water stability and solubility^32^.

In the present work, we have employed a mixed metal approach to get both water stability and activity in one single MOF system. ZIF-8 and ZIF-67 are highly compatible due to their iso-reticular structure and similar lattice parameters and thus the mixed metal ZIF is synthesized via the co-precipitation of zinc and cobalt ions with Hmim. Electron microscopic images suggest that zinc and cobalt ions cohabit uniformly in the bimetallic ZIF. The unperturbed crystal structure is confirmed using x-ray diffraction. As a consequence of the metal mixing process, the optical characteristics of mixed ZIF get altered which are evaluated using excited state emission property. The water stability of mixed ZIF has been evaluated both in the short term and for a long period of time. The pH responsive dissolution of mixed ZIF is also assessed. The photoinduced reactive oxygen species generation was monitored using DCFH assay. The antibacterial effect of mixed ZIF is evaluated using drug resistance bacterial strain. The light-induced reactivity of mixed ZIF can be employed for many fold application including therapy and pollutant degradation. However, The detailed study using variant composition of metal ions are indeed of great importance and we would like to perform these types of experiments in near future.

## Experimental Details

### Chemicals

The present study includes use of Zn (NO_3_)_2_, 6H_2_O (zinc nitrate hexahydrate) (98%, Sigma Aldrich), CoCl_2_ 6H_2_O (cobalt (II) chloride hexahydrate)((Sigma Aldrich), 2-methyl imidazole (Hmim) 99%, from Sigma Aldrich, methanol 99.8%, from RANKEM), ethanol 99.9% from ACS reagent, DMSO (dimethyl sulfoxide) from, Merck, DCFDA (dichlorodihydrofluorescein diacetate) (Calbiochem). The chemicals are used without further purification. The Milli-Q water (from Millipore) is used as a solvent for all experiments.

### Synthesis of ZIF systems

Zn_50_Co_50_ (2-methyl imidazolate)_2_ nanocrystals have been prepared by employing Zn, Co and Hmim ligand ratio as 1:1:8. The concentration of metals and ligand are chosen based on the previously reported results of mixed ZIF synthesis^33^. The synthesis has been carried out through an easy and facile route following a one-pot procedure^34^. In a typical synthesis of ZIF, metal and ligand solutions are prepared individually in the same volume of methanol. 5 mL methanolic solution has been prepared using 50 mM each of the metal salt. Ligand solution is prepared by dissolving 292 mg Hmim in 5 mL methanol. The two solutions are mixed under stirring conditions at room temperature and kept undisturbed for 24 hr. The resultant solid products are parted by centrifuging (at 5000 rpm, 5 min), washed thrice with methanol to remove excess Hmim and then dried overnight at room temperature. ZIF-8 and ZIF-67 are synthesized following the reported strategy using Zn(NO_3_) and CoCl_2_ as precursors respectively.

### Characterization

The crystallinity of the synthesized MOF is analysed using powder X-ray diffraction (XRD) using a PANalytical XPERT-PRO diffractometer equipped with Cu Kα radiation (λ = 0.15418 nm) at room temperature^19^. The samples were scanned between 5–40° 2ϴ ranges at a scan rate of 0.02° s^−1^. To characterize the morphology arrangement of the samples, transmission electron microscopy (TEM) experiment has been performed. TEM images are collected of the sample in DMSO solvent onto a carbon film-supported copper grid. The micrographs were taken at a magnification of 100000x using an FEI microscope (Tecnai S-Twin, operating at 200 kV)^35^. For optical spectroscopy measurements, absorption spectra are recorded with a Shimadzu UV-2600 spectrophotometer using a quartz cell (1 cm path length)^36^. The time resolved fluorescence transients are detected using TCSPC (time-correlated single-photon counting) setup with MCP-PMT (Edinburgh instrument, U.K.), The Instrument response function (IRF) of ~80 ps using a 375 nm excitation laser source^37^. Thermal stability is recorded under a nitrogen atmosphere and all the samples are heated from 30 to 800 °C at a rate of 10 °C/min by using a PerkinElmer TGA-50H instrument^38^.

### Aqueous stability of ZIF samples

To check, water stability of all the ZIF samples, we have synthesized ZIFs using methanol as the suitable solvent. For all the solution phase experiments, we have used dimethyl sulphoxide as the solvent. The solid samples are poured in water for a certain time and then it was collected using multiple centrifugation steps. Finally, the water treated solids are heated at 60 °C for 3 hr for drying before the XRD analysis. The water stability experiments are performed using solid ZIF samples, 2 mg each of the samples are dissolved in DMSO and 100 µL of the solution are mixed with 2 mL of distilled water. The absorbance at 595 nm is measured at 1 min time interval. Moreover, the solid samples are kept in water for 10 days at room temperature, dried and XRD is recorded. The morphology of the ZIFs is analysed using scanning electron microscopy (Quanta FEG 250: FEG source; accelerating voltage, 200 V to 30 kV; resolution).

### pH responsive dissolution

To check, the dissolution pattern of Zn_50_Co_50_-ZIF, 2 mg sample is dissolved in DMSO and 100 µL of the solution are mixed separately with 2 mL of phosphate buffer (pH = 7.4) and acetate buffer (pH = 5.5). The absorbance at 595 nm is monitored at an hour time interval.

### Dichlorofluorescein (DCFH) test for ***in vitro*** ROS generation

DCFH was prepared by composition of 0.5 ml of 1.0 mM DCFH-DA in methanol solvent with 2.0 ml of 0.01 N aqueous NaOH for 30 min at room temperature. The mixture was neutralized with 10 ml 25 mM NaH_2_PO_4_ at pH 7.4 as reported earlier^39,40^. The final solution was stored on ice in dark conditions. In this assay, DCFH oxidation leads to formation of a fluorescent DCF, which can be taken upon excitation at the wavelength of 488 nm, using steady-state fluorescence emissions. The extends in the fluorescence intensity at around 520 nm reflects the generation of ROS. For recyclability assay, fresh DCFH is added to the system after 1 hr and the assay is continued for consecutive three cycles. The standard white LED light source of 6-watt power is used to irradiate the samples.

### Bacterial assay: strain and culture conditions

The antibacterial assays have been performed using antibiotic resistant bacterial strains gram-positive methicillin-resistant *S. aureus* (MRSA). To perform the growth analysis of bacteria under different treatment conditions, bacterial culture of 12 hrs is diluted 20 times and different samples are added. The samples were incubated for 2 hr followed by light irradiation of 30 min. The absorbance at 540 nm is monitored with 30 mins interval. Only bacterial culture is considered as negative control. The absorbance is plotted against time and nature of the plot suggests relative effectiveness of the ZIF samples as an antibacterial agent. For colony forming unit assay, we have chosen 1 mg/mL concentration of sample. MRSA culture was incubated with the requisite sample for 3 hr under dark condition followed by 30 mins of white light irradiation followed by plating of the cultures. The plates were incubated for overnight at 37 °C and well grown colonies were counted for estimation of antibacterial effect of Zn_50_Co_50_-ZIF sample.

### Photocatalysis test

The photocatalytic activity of the mixed ZIF under visible light illumination was performed for photo-decomposition of methylene blue (MB), a model pollutant in aqueous solution^41,42^. The photodegradation of MB was examined in a quartz cell (1 cm optical path) containing 3 mL of the solution (1 g L^−1^) of ZIF. The suspension was irradiated under visible light using a standard white LED light source of 6-watt power and appropriate amounts of aliquots were taken out at certain time intervals.

## Results and Discussion

**Figure 1:**
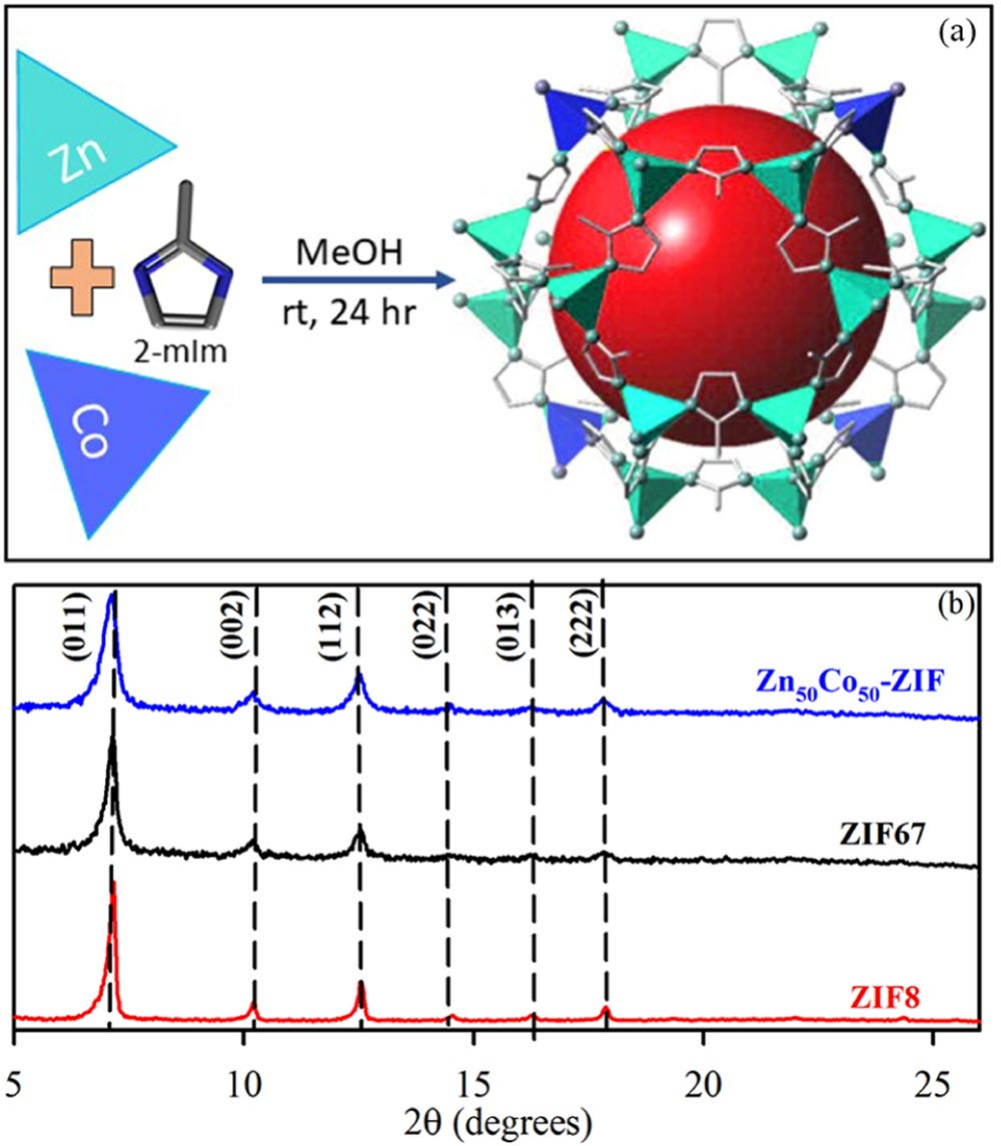
(**a**) Schematic representation of synthetic strategy for mixed metal zeolitic imidazolate framework (ZIF NPs) (**b**) XRD patterns of synthesized ZIF-8 (red), ZIF-67 (black) and bimetallic ZIF (blue).

**Figure 2:**
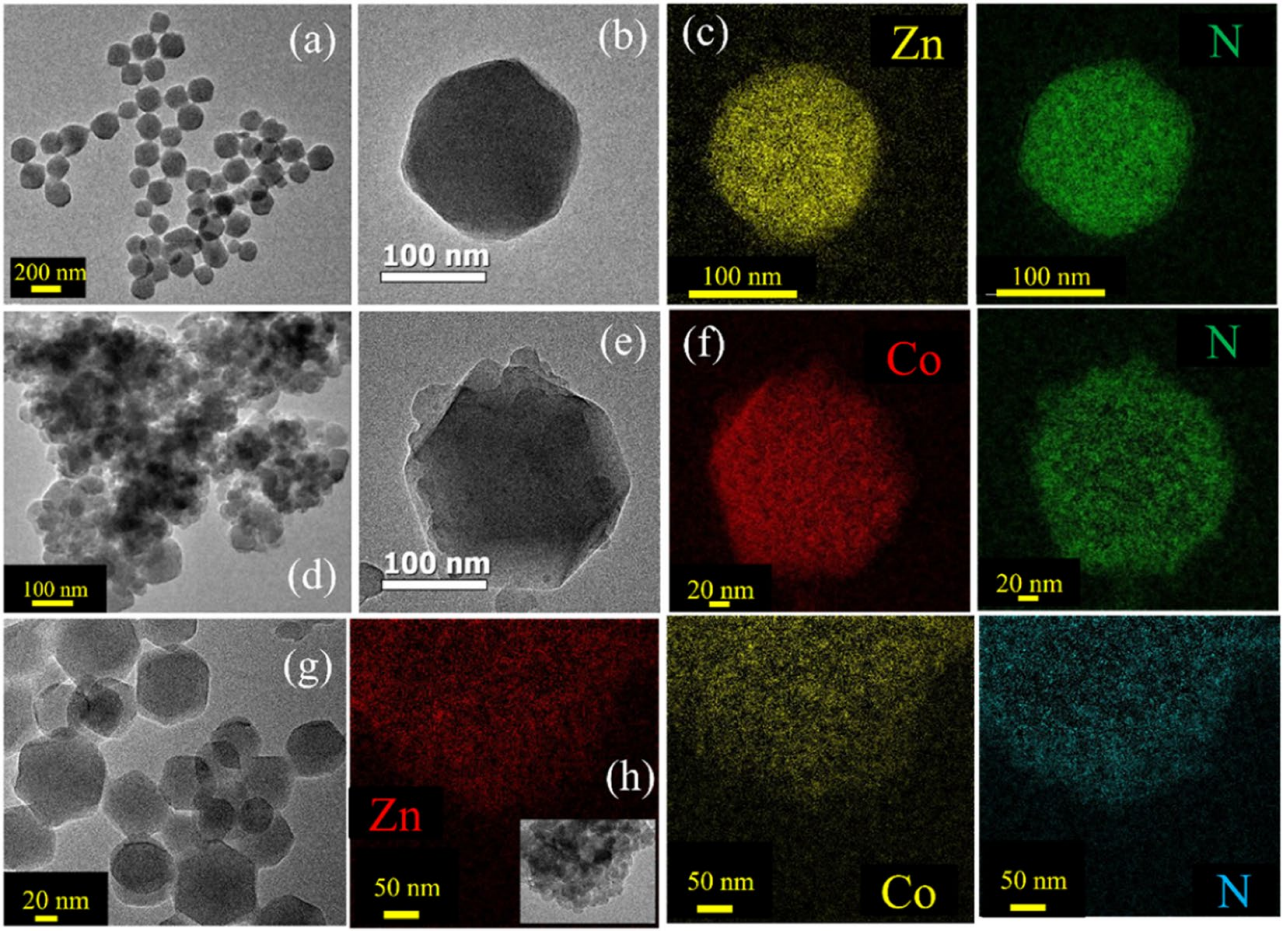
(**a**) TEM images of ZIF-8 NPs (**b**) TEM image of single nanoparticle (ZIF-67) (**c**) EFTEM compositional analysis of ZIF-8 NPs (**d**) TEM images of ZIF-67 NPs (**e**) TEM image of single nanoparticle **(f**) EFTEM compositional analysis of ZIF-67 NPs. (**g**) TEM images of Zn_50_Co_50_-ZIF NPs (**h**) EFTEM compositional analysis of Zn_50_Co_50_-ZIF NPs.

Bimetallic ZIFs are prepared by reacting Co^2+^ and Zn^2+^ metal ions with 2-methylimidazolate (Hmim) in methanolic solution as depicted in Fig. 1(a). The mixed-coordination of Hmim with an initial similar molar ratio of Zn^2+^ and Co^2+^ ions used in synthesis, have been denoted as Zn_50_Co_50_-ZIF. Figure 1(b) shows the XRD patterns of all synthesized samples namely ZIF-8 (red), ZIF-67 (black) and Zn_50_Co_50_-ZIF (blue). All samples exhibit the same peaks at 7.22°, 10.2°, 12.5° and 17.8°, which corresponds to the diffraction planes (011), (002), (112) and (222) respectively which agree with reported XRD of ZIF-8^43^. It indicates that all samples are in pure phases and isostructural with ZIF-8 showing high crystallinity. Next, we have used transmission electron microscopy (TEM) for structural characterization of these samples. TEM results revealed the size and morphology of ZIF-8 (Fig. 2a,b) and ZIF-67 (Fig. 2d,e) as uniform rhombic dodecahedral shaped crystals with an average diameter of 150–250 nm. To observe the chemical composition of ZIF systems, we have employed Energy Filtered TEM (EFTEM) mapping analysis of ZIF-8 (Fig. 2c) and ZIF-67 (Fig. 2f). The EFTEM map of a single crystal and multiple crystals of both ZIF-8 and Zn_50_Co_50_-ZIF depicts (Figs. S1 and S2) the homogeneous distribution of metal ions (Zn^2+^ and Co^2+^) and ligand atoms (N) throughout the crystal. Zn_50_Co_50_-ZIF crystals are also characterised using TEM analysis. Figure 2g depicts the single crystal morphology of the bimetallic ZIF. The average diameter of the crystals is 100 nm. The EFTEM map (Fig. 2h) shows homogeneous distribution of metal ions (Zn^2+^ and Co^2+^) and ligand atoms (N) throughout the crystal. Quantitative analysis of Zn and Co K shell maps collected using the energy dispersive X-ray (EDX) elemental mapping analysis (data not shown) depicts that the weight percentages of Zn and Co are 11.8% and 10.73% respectively. The observation suggests similarity between the ratio of Co and Zn used in the synthesis with the metal loaded within the crystal, indicating formation of actual Zn_50_Co_50_-ZIF crystals.

**Figure 3:**
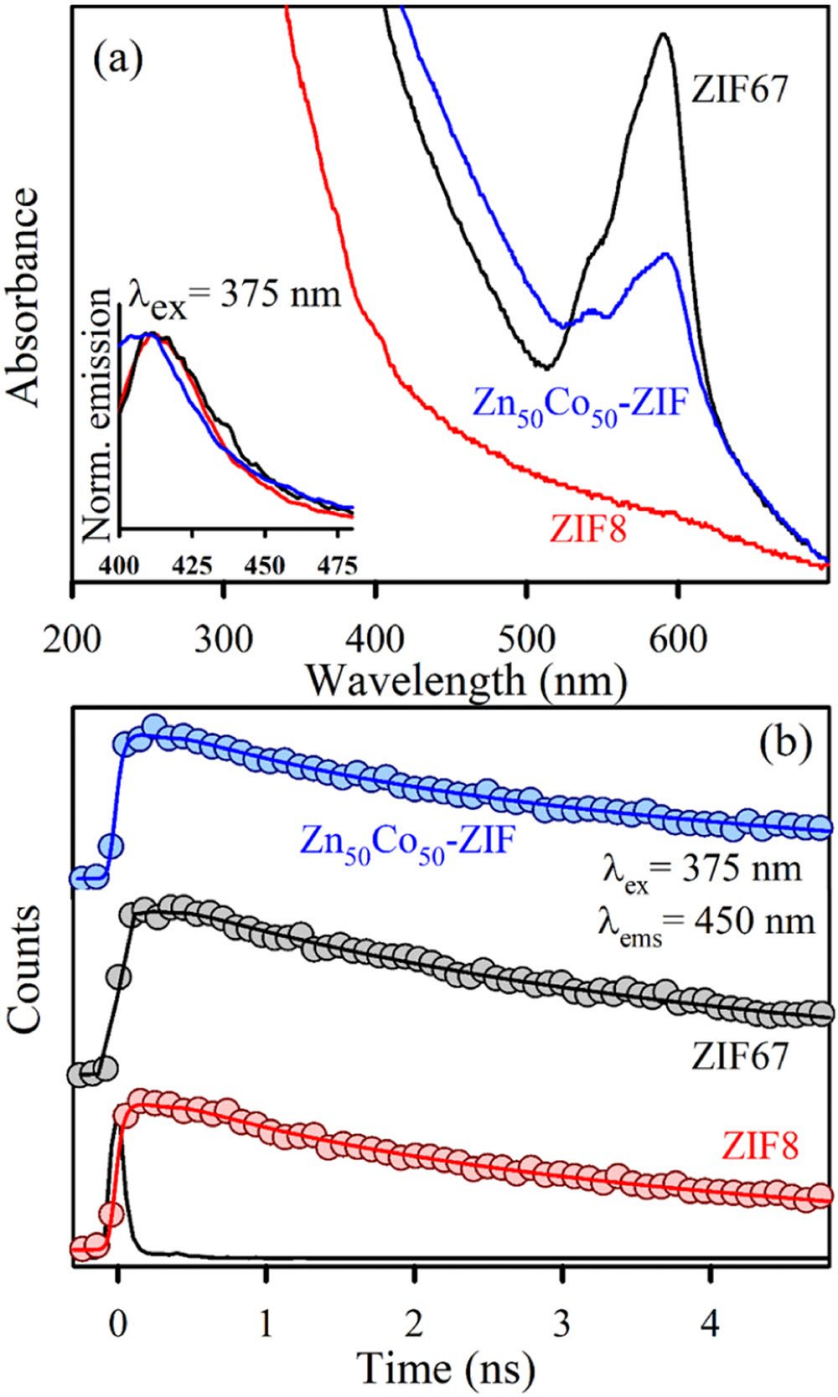
(**a**) UV-Vis absorption spectra of ZIF-8 (red), ZIF-67 (black) and Zn_50_Co_50_-ZIF (blue). Inset shows the corresponding normalized emission spectra upon excitation at 375 nm. (**b**) Picosecond resolved fluorescence transients of ZIF-8 (red), ZIF-67 (black) and Zn_50_Co_50_-ZIF (blue) collected at 450 nm upon excitation at 375 nm.

After the structural characterizations the optical absorption spectra of the ZIF systems are evaluated. The absorption spectrum of ZIF-8 is located in the UV region and is virtually featureless in the visible region. ZIF-67 attributes absorption maxima at 588 nm with two shoulder peaks at 565 nm and 537 nm (Fig. 3a). These absorption characteristics generate due to the higher-lying [4A2(F) to 4T1(P)] d-d ligand field transitions^44^. The observation also suggests presence of Co^2+^ in a tetrahedral environment. Following the mixed metal ZIF, the absorption characteristics are like that of ZIF-67 with a slight red shift in the peak position. The main absorption maximum is at 593 nm with shouldered at 570 nm and 540 nm. All the ZIF system show broad absorption characteristics in <400 nm region due to the presence of ligand to metal charge transfer (LMCT) processes. Although the mixed metal ZIF is isostructural with that of ZIF-67, it possesses decreased absorption of Co d–d transitions owing to the dilution of the Co^2+^ chromophores by the non-absorbing Zn^2+^ ions^45^. The evaluation of photoluminescence property of the ZIF systems suggests that all three ZIFs provide emission maximum around 450 nm upon excitation at 375 nm (lower inset of Fig. 3a). The emission band arises mainly due to the association of linker with the coordinating metal ions and remains at the same position for various metal ions i.e. Zn^2+^ (d^10^) or Co^2+^ (d^7^)^46^. We have then performed the excited state photoluminescence experiments to unravel the charge transfer process. The fluorescence decay profiles for ZIF-8, ZIF-67 and Zn_50_Co_50_-ZIF were obtained upon photoexcitation at 375 nm in ethanol and monitored at 450 nm as shown in Fig. 3b. The time profile of the fluorescence decay at 450 nm for ZIF-8, ZIF-67 and Zn_50_Co_50_-ZIF showed biexponential decay, with a faster component of ~800 ps and a slower component of 4 ns. The lifetime components of the transients are represented in Table 1. As evident from Table 1, time components appear to be similar in all the fluorescence transients indicating superior involvement of ligand for the emission characteristics.

**Table 1:** Picosecond-resolved fluorescence transient lifetime.

System	τ_1_ (ps)	τ_2_ (ps)	τ_avg_ (ps)
ZIF-8	847 (17%)	4462 (83%)	3847
ZIF-67	730 (7%)	4212 (93%)	3968
Zn_50_Co_50_-ZIF	791 (17%)	4440 (83%)	3819

The emission (monitored at 450 nm) was detected with 375 nm laser excitation. Numbers in parentheses indicate relative contributions.

**Figure 4:**
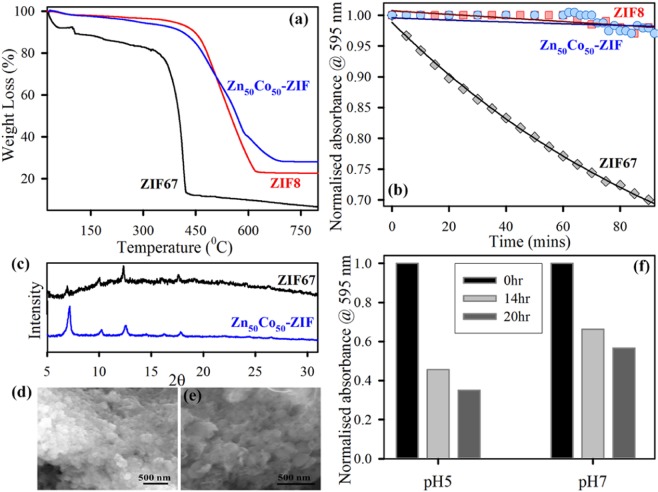
(**a**) Thermogravimetric profile of ZIF-8 (red), ZIF-67 (black) and Zn_50_Co_50_-ZIF (blue) monitored under N_2_ flow. (**b**) Aqueous stability of ZIF-8 (red), ZIF-67 (black) and Zn_50_Co_50_-ZIF (blue) monitored using absorption at 595 nm for 90 mins time frame. (**c**) XRD pattern of ZIF-67 (black) and Zn_50_Co_50_-ZIF (blue) after keeping in water for 10 days at room temperature. SEM images of (**d**) ZIF-67 and (**e)** Zn_50_Co_50_-ZIF, after keeping in water for 10 days at room temperature. (**f**) Dissolution pattern of Zn_50_Co_50_-ZIF at two different pH buffer solutions (pH = 5 and 7 respectively) over different time intervals as 0 hr (black), 14 hr (light grey), 20 hr (dark grey).

The thermal behavior of the ZIFs is examined via thermogravimetric analysis (TGA). Figure 4a shows the thermogravimetric (TG) curves of ZIF-8, ZIF-67 and Zn_50_Co_50_-ZIF implementing the increasing temperature method. The first weight loss of ZIF-67 occurs from 100 °C to 200 °C due to the disappearance of guest molecules as well as gas molecules from the cavities. Subsequently, a more weight loss of ZIF-67 occurred in the range of 300–450 °C, which could be imposed to the putrefaction of the overall crystal structure. For ZIF-8 sample, an unchanging weight loss profile is reached with respect to temperatures up to 450 °C, upon which effective weight loss occurs with respect to temperature and suggests the onset of impermanence temperature of the ZIF-8 nanoparticles. After reaching either rottenness temperature, it indicates a steep reduction in weight analogous to a downfall of the ZIF-8 structure and carbonization under extreme thermal stress^47^. The bimetallic ZIF depicts similar thermal behaviour with ZIF-8 system. There is only one step weight loss is associated which is due to the decomposition of the organic linker. It has been observed that Zn_50_Co_50_-ZIF is quite stable and begins to decompose at 450 °C. Apart from the TGA, we performed XRD tests of Zn_50_Co_50_-ZIF before and after the heat treatment to the sample. The XRD peaks of Zn_50_Co_50_-ZIF before heat is exactly similar to XRD peaks with after heated at 160 °C for 1 hour as shown in Fig. S3. The observation suggests that incorporation of Zn(II) in ZIF-67 crystal improves its thermal stability probably due to that Zn(II) ion has a stronger coordination capability to 2-methylimidazolate than Co(II) ion^48^. As the mixed metallic Zn_50_Co_50_-ZIF nanoparticles show improvement in thermal stability, so we have assessed its aqueous stability in short time frame as well as in long term. Figure 4b shows the change in absorbance at 595 nm (main peak of ZIF systems) with increasing time. It has been clearly observed that ZIF-8 is very stable without unaltered absorbance value after 90 mins, but ZIF-67 shows ~30% decrease in absorbance value. This suggests that ZIF-67 is not stable in aqueous environment owing to the spontaneous hydrolysis of ZIF-67 due to breaking of weak Co-Imidazolate bond. Zn_50_Co_50_-ZIF shows typical stability in water with almost no change in absorbance value at 595 nm. The improved water stability may arise due to lesser possibility of crystal hydrolysis as the Zn-Imidazolate bond is much stronger. Further, the long-term water stability of the systems is analyzed using XRD and SEM analysis. XRD pattern of ZIF-67 shows complete perturbation of crystal structure after 10 days of water exposure, whereas the XRD signals remain unchanged for the Zn_50_Co_50_-ZIF under the same condition (Fig. 4c). The SEM images of Zn_50_Co_50_-ZIF and ZIF-67 after 10 days of water exposure are shown in Fig. 4d,e respectively. The SEM images also confirm the total structural destruction of ZIF-67 upon long term water exposure which gets improved in case of Zn_50_Co_50_-ZIF systems^44^. The thermally stable and water steady Zn_50_Co_50_-ZIF nanoparticles are finally evaluated for its pH responsive deposition nature. The decrease in the absorbance at 595 nm corresponds to the degree of dissolution of Zn_50_Co_50_-ZIF. Figure 4f shows that there is ~30% increase in dissolution at acidic pH (pH-5.5) compare to physiological pH (pH-7.4). The pH responsive dissolution trend is mainly because ZIF is a porous network assembled by the coordination interactions within ligand (methylimidazole) linkers and metal ions. The acidic environment nature will protonate methylimidazole (pKa ~ 7.35) that may decrease the coordination interactions and this leads to a damaged network^48^. As it has been already reported that Zn-N bond in ZIF-8 tends to dissociate in acidic pH, so the trend can be observed in the mixed system also. The enhanced stability (both thermal and aqueous), as well as the pH responsive dissolution of Zn_50_Co_50_-ZIF, indicates its superior potential for various application purposes. We have chosen and analyze its light-induced effectivity as an alternative antimicrobial agent and a photocatalyst.

**Figure 5:**
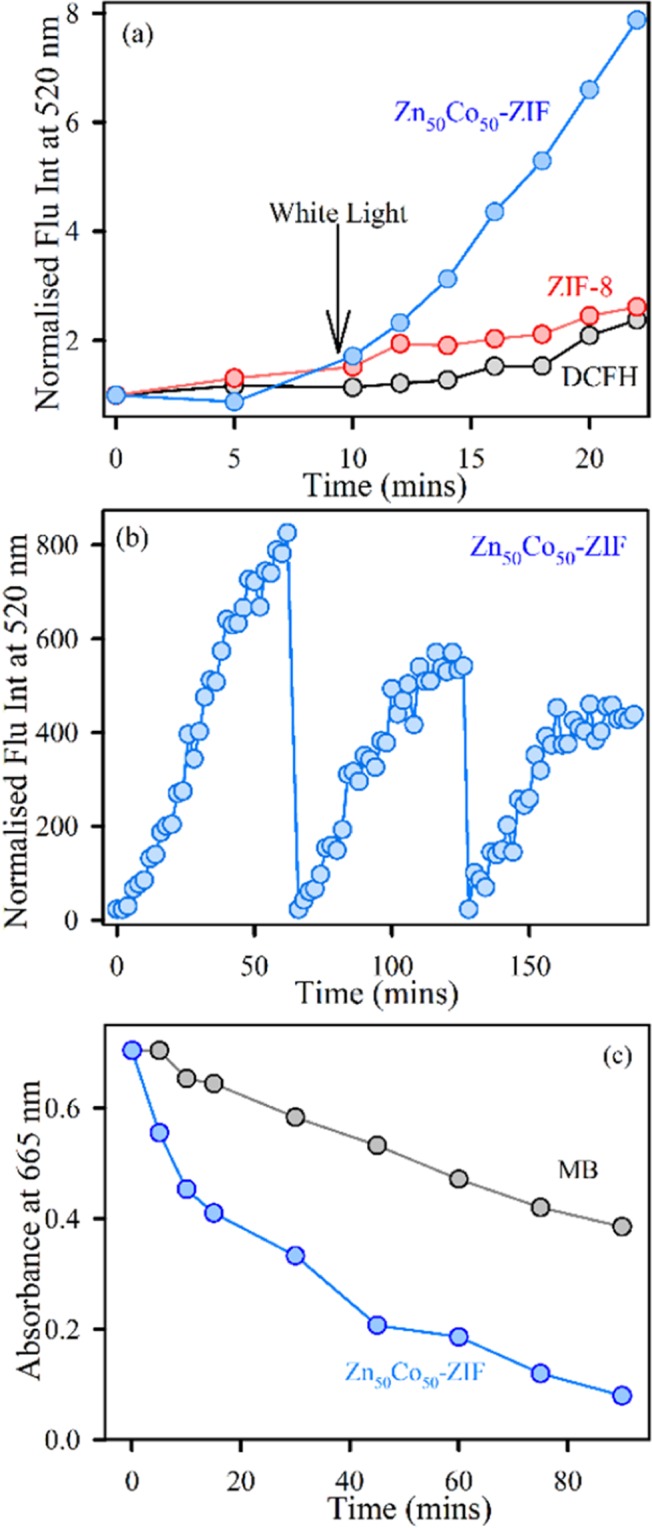
(**a**) DCFH oxidation with respect to time with addition of ZIF-8 (red), Zn_50_Co_50_-ZIF (blue) and DCFH control (black) under dark and subsequent white-light irradiation. (**b**) Recyclability of ROS generation by Zn_50_Co_50_-ZIF under white light irradiation monitored by DCF fluorescence at 520 nm. (**c**) Kinetics of methylene blue degradation under white light illumination (control: grey, Zn_50_Co_50_-ZIF: blue).

We have evaluated the photoinduced reactive oxygen species generation by Zn_50_Co_50_-ZIF as the sample possesses broad absorption characteristics, as well as some recent reports, suggest there exists a stable higher energy state in ZIF-67 which can generate reactive species. The increase of DCF emission upon oxidation of DCFH by ROS has been monitored to detect the amount of ROS. Zn_50_Co_50_-ZIF nanoparticle shows 2.5 times more ROS generation efficiency compare to ZIF-8 under white light illuminated condition as depicted in Fig. 5a. The ROS generation capability is also recyclable up to three consecutive cycles (Fig. 5b). The fitted curve with exponential rise equation depicts that rate constant for 1st cycle is 186 min. Thereafter rate constants for 2nd and 3rd cycles are 48 min and 40 min respectively. As depicted by transient absorption spectroscopy, there exists a long-lived intermediate excited state in ZIF-67 systems^44^. Thus, we have predicted that mixed ZIF also possesses such stable excited electronic states which contribute to ROS formation ability. We have also performed photocatalysis of model pollutant MB to check environmental aspect of mixed ZIF system Fig. 5c shows the kinetics of degradation of MB that depicts 75% degradation in 90 mins. The reactive species such as ∙OH and O_2_∙ are engaged in the photocatalytic oxidation process. Hence, the effects of radical scavengers on the photo degradation of MB were performed to unravel the reaction process. as the scavenger of ∙OH, TBA (Tertiary butyl alcohol) was introduced. The degradation efficiency of Zn_50_Co_50_-ZIF photocatalyst for MB is reduced to 30% after adding TBA and decreased to 5% after adding N_2_. Therefore, It can be clearly seen that ∙OH redicals have a significant role in the photocatalytic oxidation process of MB, whereas O_2_∙ has a slight effect on this mechanism. We have depicted in the supporting information Fig. S4. Apart from that, in order to confirm check the metal leaching during photocatalysis, We did colorimetric test and EDAX test (Fig. S5), after photocatalysis of filtered liquid solutions. Also, the XRD experiment of Zn_50_Co_50_-ZIF has been performed before and after photocatalysis. The XRD peaks of normal Zn_50_Co_50_-ZIF are exactly similar to the XRD peaks of Zn_50_Co_50_-ZIF after photocatalysis as shown in supporting information (Fig. S6). Above tests are sufficiently justified our conclusion that metal leaching is not happening during the photocatalysis.

**Figure 6:**
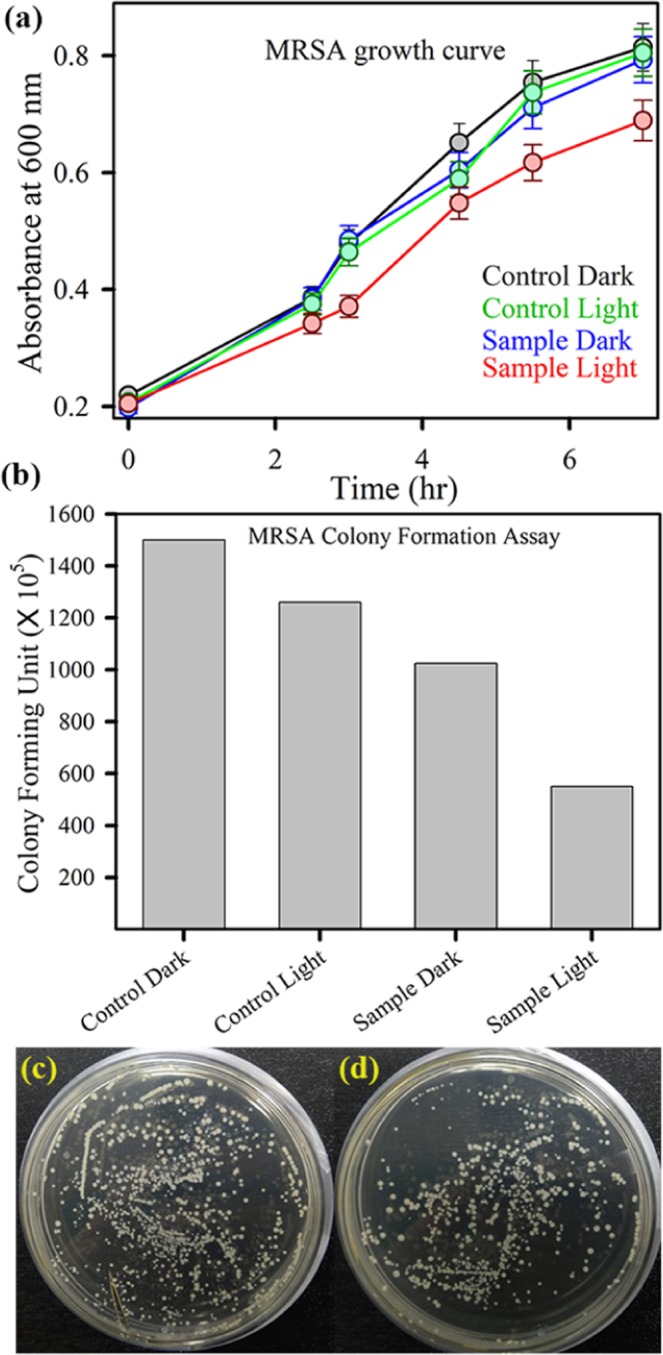
(**a**) Optical density measurement for Methicillin resistant *S. aureus* grown in the presence of Zn_50_Co_50_-ZIF under dark (blue) and white light (red) (control dark is black, and control light is green curve). The error bars represent the standard deviation of three independent measurements. The lines connecting the experimental points are for visual aid. (**b**) Bacterial viability after treatment with Zn_50_Co_50_-ZIF in the presence and absence of white light irradiation. (**c**) Shows images of MRSA plates treated with Zn_50_Co_50_-ZIF, before (left) and after (right) white light irradiation.

Finally, we have evaluated the antibacterial action of Zn_50_Co_50_-ZIF using clinically relevant drug resistant bacterial strain gram-positive methicillin resistant *Staphylococcus aureus* (MRSA). Figure 6a shows the MRSA growth curve upon treatment with Zn_50_Co_50_-ZIF. The effect of Zn_50_Co_50_-ZIF on the bacteria-growth kinetics experiment in LB aqueous media showed a typical t antibacterial effect in the presence of visible light. Compared to the growth curve of the control MRSA bacteria, the presence of mixed ZIF samples can slow the growth of MRSA. It is reasonable to determine that the executed high and long-term photodynamic antibacterial activity of Zn_50_Co_50_-ZIF is due to their higher stability than that of ZIF-67. We have also performed the colony formation assay to check the phototoxicity. As shown in Fig. 6b, it is clearly observed that Zn_50_Co_50_-ZIF destroys MRSA colonies around 45% more in presence of light than the dark condition. Figure 6c,d show the respective images of plates that show the distinct change in number of colonies after light treatment. The sustained dissolution at acidic pH following release of metal ions and synergistic effects of dual metal ions uptake by the bacterial cells promotes cell death. Overall, the greater water stability and ROS generation employ a new way for different types of applications of mixed metallic MOF as Zn_50_Co_50_-ZIF systems like photodynamic therapy, photocatalysis etc.

## Conclusion

The present study employs a synthesis of novel mixed metal zeolitic imidazolate framework to enhance the applicability of MOF based systems. The synthesis follows solution phase strategy and an equal molar ratio of Zn and Co metal ions are incorporated within the framework using imidazolate as the linker. The Zn_50_Co_50_-ZIF nanocrystals have been structurally characterized using XRD and TEM. Energy filtered mapping of the crystal suggest homogeneous distribution of Zn and Co ions. The optical absorption spectra confirm that incorporation of Co improves the visible light absorption property. The photoluminescence analysis suggests that emission peak at 450 nm in ZIF system mainly originated from the ligand part and the excited state lifetime is independent of the metal characteristics. The mixed metal shows enhanced thermal stability, water stability and pH responsive dissolution characteristics. The reason behind improvement of the properties is mainly the synergistic effect between the two metal ions. Zn is mainly responsible for stability whereas Co improves the activity. The generation of photoinduced reactive oxygen species (ROS) by Zn_50_Co_50_-ZIF under white light illumination is monitored. The ROS generation is found to be recyclable. Zn_50_Co_50_-ZIF depicts antibacterial photodynamic therapeutic activity towards drug-resistant bacterial strains. It can also degrade model pollutant MB under white light illumination. The overall investigation implements mixed ZIF as potential biomedicine with great biocidal property and also as a future light-harvesting material for manifold application.

## Supplementary information

Supporting Information
