# Supplementary material for: Enhanced Water Stability and Photoresponsivity in Metal-Organic Framework (MOF): A Potential Tool to Combat Drug-resistant Bacteria
Source: Sci Rep. 2019 Dec 18;9:19372. doi: 10.1038/s41598-019-55542-8 (PMC6920456; doi:10.1038/s41598-019-55542-8)
Supplement: Supplementary file 1 — Supporting Information [file 41598_2019_55542_MOESM1_ESM.docx]

**Supporting Information**

**Enhanced Water Stability and Photoresponsivity in Metal-Organic Framework (MOF): A Potential Tool to Combat Drug-resistant Bacteria**

Saleh A. Ahmed, *^, [a,b]^ Damayanti Bagchi,^[c]^ Hanadi A. Katouah,^[b]^ Md. Nur Hasan, ^[c]^ Hatem M. Altass, ^[b]^ Samir Kumar Pal*^, [c]^

*^a^Chemistry Department, Faculty of Applied Sciences,*

*Umm Al-Qura University, 21955 Makkah, Saudi Arabia*

*^b^Chemistry Department, Faculty of Science, Assiut University, 71516 Assiut, Egypt*

*^c^Department of Chemical, Biological and Macromolecular Sciences,*

*S. N. Bose National Centre for Basic Sciences,*

*Block JD, Sector III, SaltLake,*

*Kolkata 700 106, India*

^*^Corresponding Authors

Email: [skpal@bose.res.in](mailto:skpal@bose.res.in), [saahmed@uqu.edu.sa](mailto:saahmed@uqu.edu.sa), [saleh_63@hotmail.com](mailto:saleh_63@hotmail.com)


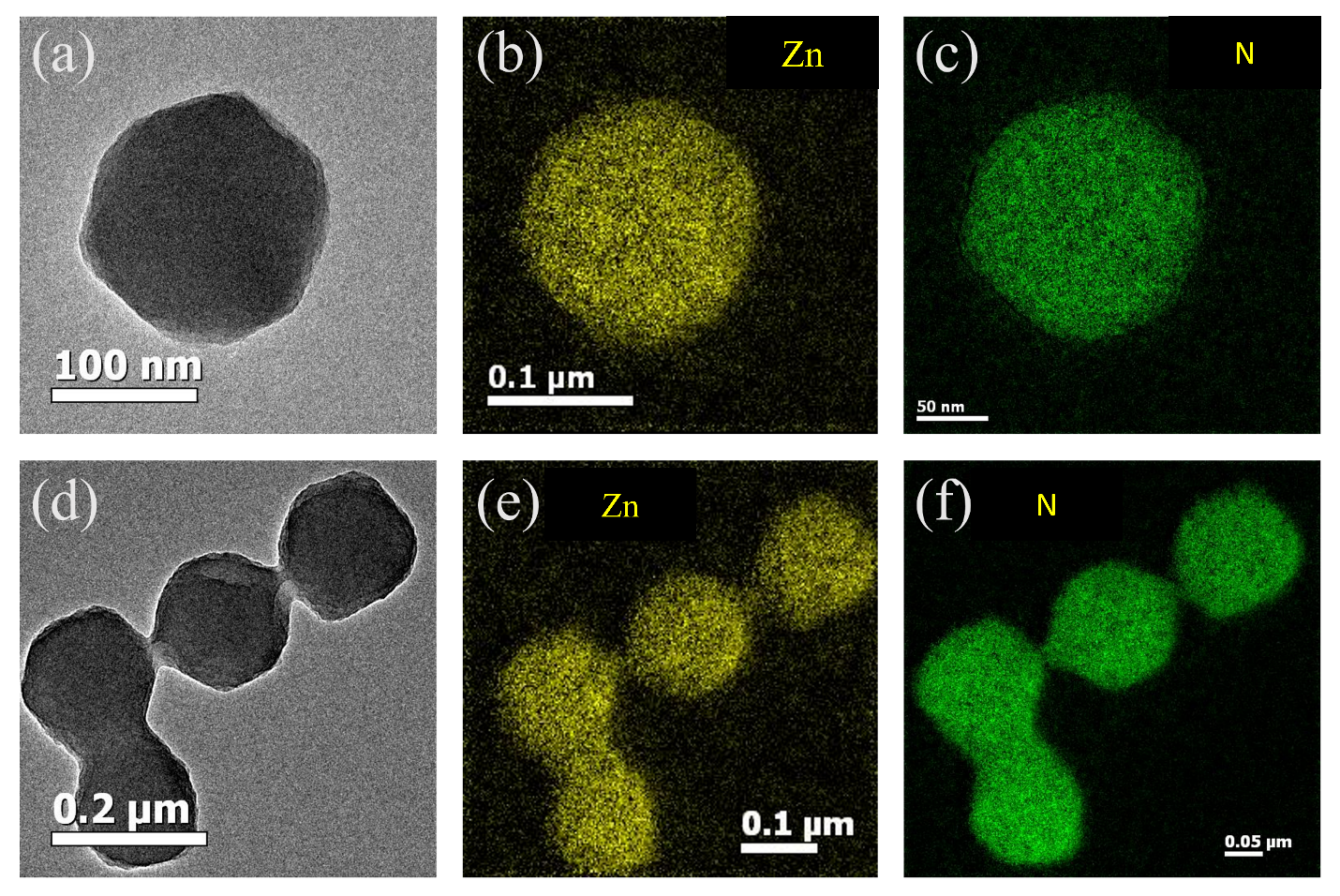


**Figure S_1_:** EDAX mapping of ZIF-8.


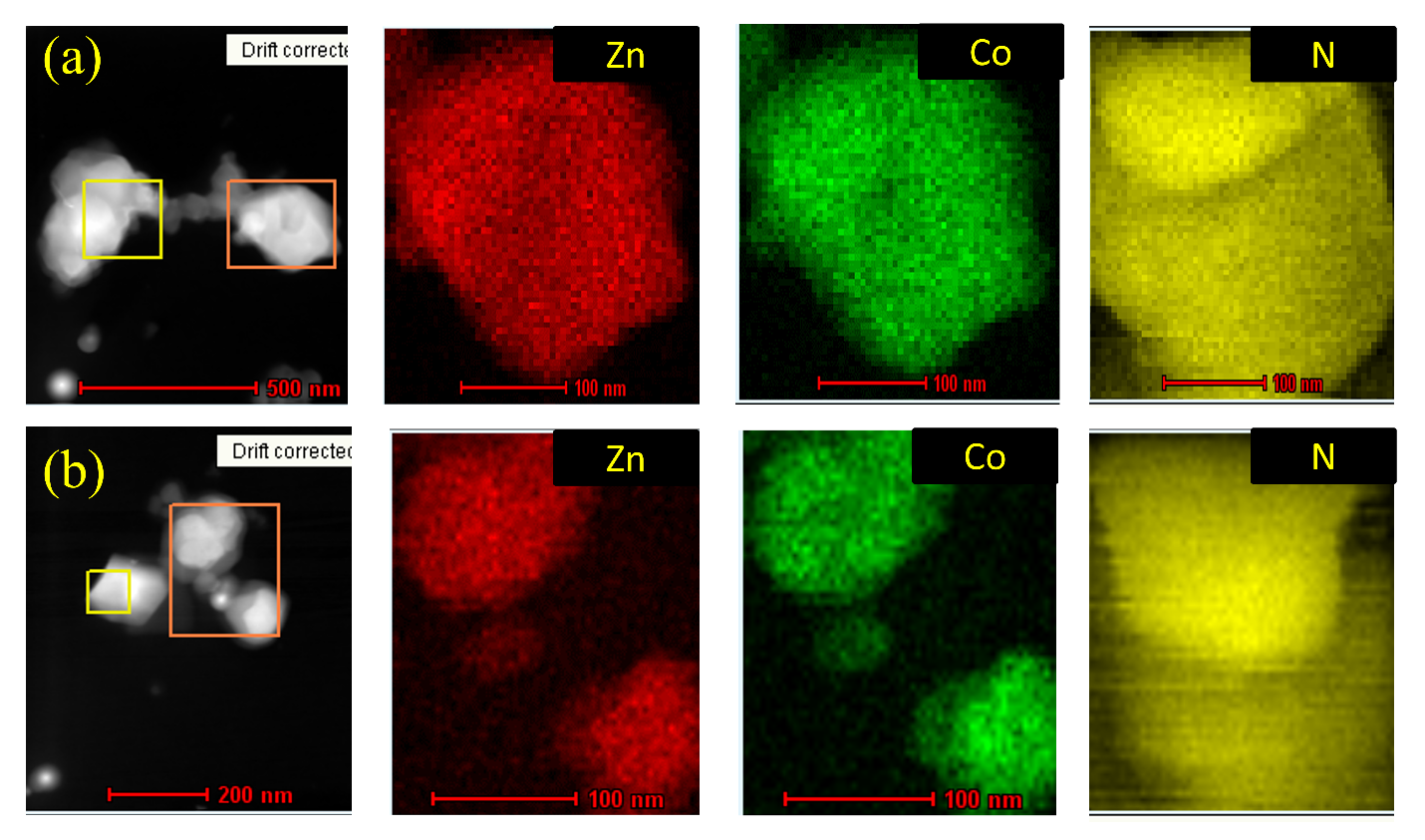


**Figure S_2_:** EDAX mapping of Zn_50_Co_50_-ZIF.





**Figure S_3_:** XRD experiment of Zn_50_Co_50_-ZIF before heat (blue) and after heated at 160 °C upto 1 hour (red).





**Figure S_4_:** Effect of N_2_ and TBA on the photocatalytic activity of Zn_50_Co_50_-ZIF.





**Figure S_5_:** EDAX of filtered liquid solution.





**Figure S_6_:** XRD experiment of Zn_50_Co_50_-ZIF before photocatalysis (blue) and after photocatalysis (red).
